# Supplementary material for: “A Huge Gap”: Health Care Provider Perspectives on Cancer Screening for Aboriginal and Torres Strait Islander People in the Northern Territory
Source: Int J Environ Res Public Health. 2024 Jan 27;21(2):141. doi: 10.3390/ijerph21020141 (PMC10887611; doi:10.3390/ijerph21020141)
Supplement: Supplementary file 1 [file ijerph-21-00141-s001.zip › ijerph-2806764-supplementary.pdf]

Western Australian Centre for Rural Health

THE UNIVERSITY OF WESTERN AUSTRALIA

Exploring innovations and consumer and service provider perspectives of Indigenous cancer care delivery and support in the Northern Territory

Interview Guide - Health Professionals

General areas to be discussed:

1. Could you tell me about your role in delivering cancer treatment to Aboriginal and Torres Strait Islander patients? (Specialty, number of years practicing, catchment area, role, etc.)
2. What is the typical treatment pathway (patient journey) for patients requiring treatment? (If appropriate - please describe how that differs from 10 years ago)
3. Please describe how decisions about cancer treatment options are generally made? Are patients involved in the decision-making? How are they involved?
4. What are your views on the quality of the services provided by the Cancer Centre/Hospital/Clinic where you work to Aboriginal and Torres Strait Islander patients? (and compared with other service centres) in terms of the following:
   a. Consults with Aboriginal and Torres Strait Islander stakeholders to establish and run programs/services
   b. Establishes links with primary care providers and integration/continuity of services
   c. Identifies distinctive care needs of Aboriginal and Torres Strait Islander patients and responds to enable better support, e.g. logistical support, psycho-social support, telehealth, etc.
   d. Assesses improvements in cancer outcomes and quality of life (QOL) for Aboriginal and Torres Strait Islander patients
   e. Provides appropriate information and resources for Aboriginal and Torres Strait Islander people affected by cancer around cancer and cancer care
   f. Engages in primary prevention and Aboriginal and Torres Strait Islander community education around cancer and cancer care.
5. How could the Cancer Centre/Hospital/Clinic where you work improve to better meet the needs of Aboriginal and Torres Strait Islander patients? And what changes could improve cancer services generally – do you have any suggestions?
6. Can you describe any barriers (e.g. financial, logistical, etc.) you are aware of for Aboriginal and Torres Strait Islander people accessing treatment at this Cancer Centre/Hospital/Clinic?
7. Is there anything else you would like to add?
